# The role of alerting in the attentional boost effect

**DOI:** 10.3389/fpsyg.2023.1075979

**Published:** 2023-04-06

**Authors:** Fajie Huang, Guyang Lin, Yingfang Meng, Yuanyuan Lin, Siqi Zheng

**Affiliations:** ^1^School of Health, Fujian Medical University, Fuzhou, China; ^2^School of Psychology, Fujian Normal University, Fuzhou, China; ^3^Education Research Institution of Fujian Province, Fuzhou, China

**Keywords:** dual-task, target detection, memory, attentional boost, alerting

## Abstract

Stimuli presented simultaneously with behaviorally relevant events (e.g., targets) are better memorized, an unusual effect defined as the attentional boost effect (ABE). We hypothesized that all types of behaviorally relevant events, including attentional cues, can promote the encoding process for the stimuli paired with them, and the attentional alerting network can amplify the ABE. The two experiments we conducted demonstrated that not all behaviorally relevant events, including alerting cues, benefit the processing of concurrently paired stimuli. We also found that the presence of a cue prior to a target can extend the memory advantage produced by target detection, but this advantage can only be observed within a limited range of time. Overall, our study provides the first evidence that the alerting network plays an important role in the ABE.

## Introduction

Decades of work on dual-task performance and selective attention have provided robust evidence that dividing attention during encoding generally impairs subsequent memory performance (Kinchla, 1992; Pashler, 1994). However, recent studies have shown that detecting targets in a secondary task while simultaneously encoding a series of images into memory might actually enhance subjects’ performance on subsequent memory tests (Lin et al., 2010; Swallow and Jiang, 2010; Mulligan et al., 2014; Mulligan and Spataro, 2015).

Swallow and Jiang (2010) conducted the initial experiment that led to this unusual finding. During encoding, a series of scenes were presented on a screen and a small square superimposed at the center of each. Participants were asked to complete two tasks at the same time: remembering the scene and responding accordingly to the color of the square (white as the target and black as the distractor). Participants were asked to quickly press the button when a white square was shown and avoid doing so when the square was black. Then, the participants were asked to perform a four-alternative forced-choice recognition test. Since a greater attentional demand is required when detecting a target than when ignoring a distractor (Dux and Marois, 2009), detecting white squares (i.e., targets) should have impaired the encoding of the background scenes. However, an opposite result was observed (i.e., scenes with white squares were more accurately memorized than those with black squares), a phenomenon referred as the attentional boost effect (ABE). In another experiment conducted for the same study, when participants were instructed to memorize the scenes and ignore the squares under the full attention condition, no memory enhancement was found; scenes with target and distractor squares were equally well memorized. A later study again confirmed that a response to the target square superimposed on each scene was required for the ABE to emerge (Swallow and Jiang, 2011). It was also shown that the ABE could be obtained even when the targets were not rare (Swallow and Jiang, 2012), detected without overt motor responses (Swallow and Jiang, 2012; 2014), or perceptually similar to the distractors (Swallow and Jiang, 2014). These findings indicate that the boost is linked to target detection, which enhances the encoding and memory of target-paired but unrelated background stimuli.

The ABE effect is a surprising finding showing that the processing of unrelated background stimuli is enhanced when behaviorally relevant events occur, a conclusion contrary to the traditional negative perception of dual tasking. A possible explanation for this phenomenon is that behaviorally relevant events may lead to a processing enhancement of the broader context (Bouret and Sara, 2005; Zacks et al., 2007). Many studies have shown that perceptual and conceptual information presented simultaneously with changing observation activities is an important component of long-term memory (Lassiter et al., 1988; Schwan and Garsoffky, 2004; Swallow et al., 2009). One might then ask whether alerting cues enhance memory and the ABE. An alerting cue is a stimulus that signals that a target is forthcoming, but the cue itself does not require a response (Leclercq and Seitz, 2012). To address this question, the current study compared the recognition accuracy of stimuli presented with an alerting cue during encoding to stimuli that were not presented with such a cue; also compared were the recognition accuracy of target-paired words preceded by a cue to that of target-paired words not preceded by a cue.

Another implication of cue setting is the role of the attentional alerting system in the ABE. A well-known finding is that the attention system can be further broken down into three distinct attentional networks: alerting, orienting, and executive control (Posner and Petersen, 1990). These networks carry out independent functions (Fan et al., 2002) and are linked to isolated neural structures in the brain (Fan et al., 2005). Alerting refers to an alert state that an individual achieves and maintains to deal with external inputs; it is associated with the locus coeruleus norepinephrine (LC-NE) system (Posner and Petersen, 1990; Marrocco et al., 1994). Orienting refers to the selection of information from a variety of sensory inputs and is associated with the acetylcholine system (Davidson and Marrocco, 2000). Executive control refers to the ability to coordinate and optimize various cognitive processes to complete complex cognitive tasks with a control mechanism and is associated with the dopamine system (Fossella et al., 2002). To date, the role of these three attentional networks in the ABE is still unknown. Swallow and Jiang (2011) explored the relationship between the alerting system and the ABE. In their research, a square (which was the target) was presented either temporally overlapped with the image (the temporally overlapped condition) or 100 ms earlier (the square-early condition). Since attentional alerting can enhance perceptual processing and the effect of alerting peaks approximately 100 to 200 ms after the target onset (Nakayama and Mackeben, 1989; Egeth and Yantis, 1997; Olivers and Meeter, 2008), the images under the square-early condition should have been better memorized than those under the temporally overlapped condition. However, Swallow and Jiang (2011) did not find any memory difference between two conditions, and thus argued that alerting played a limited role in the ABE. Yet, in that study (Swallow and Jiang, 2011), the target and image did not overlap in time under the square-early condition. Several studies have shown that the temporal overlap of background stimuli and the target is a prerequisite for the ABE (Swallow and Jiang, 2010; 2011). Accordingly, it is unclear whether the lack of difference between the two conditions was the result of asynchrony in the presentation of images and targets. To address this question, the current study used a cue prior to the target, which facilitated the subject entering a more alert state as compared to the no-cue condition (Fan et al., 2005). As such, this allowed us to compare two types of target-paired words, in which the target and words were not separated temporally in varying degrees of the subject’s alert state; thus, we were better able to explore the role of the alerting network in the ABE.

## Experiment 1

### Experiment objective

In our first experiment, we tested how a visual cue presented prior to the target might impact the ABE. We used the ABE paradigm (Swallow and Jiang, 2012), however, participants encoded words incidentally, so as to exclude the possible influence of cognitive effort (as much as possible) and to more purely observe the direct influence of behavior-related stimuli (cues) on irrelevant information processing. Each word was presented with one of three types of detection stimulus: a target, a distractor, or a cue. The cue was always presented prior to the target. We expected that memory of cue- and target-paired words would be enhanced relative to the distractor-paired words. Moreover, we hypothesized that this alerting cue would further promote the memory enhancement triggered by target detection.

### Materials and methods

#### Participants

As per previous studies (Mulligan et al., 2014; Mulligan and Spataro, 2015), the average effect size of the ABE was *f* = 0.48 (equivalent to η_*p*_^2^ = 0. 19). This served as the *a priori* effect size. G*Power 3.1 was used to calculate the minimum of ten participants needed for this experiment to achieve a statistical power of 0.95 in a repeated measures analysis of variance (ANOVA) (Faul et al., 2007). Twenty-eight undergraduate students (mean age 20.3 ± 1.58 years) at Fujian Normal University participated in this experiment. All participants claimed to be in good health, have normal or corrected-to-normal vision, and no history of neurological illness. All gave their informed consent. As our objective was to study the role of the cue, we included only those participants who successfully withheld responses to the cue. Thus, subjects with more than 30% of responses to the cue in all experiments were excluded. Two subjects were excluded, and thus a total of 26 participants were included in Experiment 1.

#### Design and materials

Two attention conditions (Cue Condition vs. No-Cue Condition) were presented to each participant. A total of 287 critical words were selected from the Modern Chinese Frequency Dictionary Beijing Language College Language Instruction Institute (1986 edition): 120 critical words and 167 non-critical words. A total of 120 words were randomly divided into six sets of 20, such that the frequency, stroke, pronunciation, and configuration of the words were evenly distributed. Five sets were assigned to two encoding conditions: the Cue Condition with target, distractor, and cue, and the No-Cue Condition with target and distractor. The remaining set was not presented during the encoding phase and appeared in the test as unstudied items. Additionally, 167 non-critical words were used as filler items during the encoding phase, all these words were presented with a green square.

#### Apparatus and procedure

**FIGURE 1:**
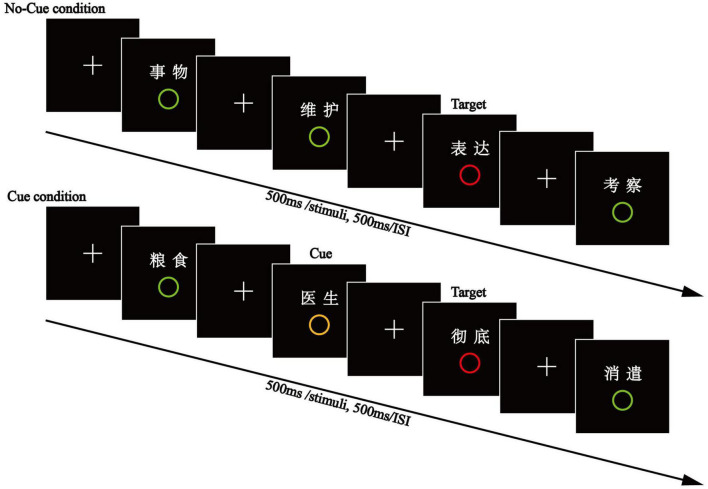
The encoding phase of Experiment 1. In each trial, a word and circle (red, yellow, or green) appeared simultaneously at the center of the screen for 100 ms. Then, the circle was removed and the word shown continuously for another 400 ms. Participants read the words and rapidly pressed the space bar key when they detected a target red circle below the word.

The experiment consisted of two consecutive phases. In the encoding phase (see Figure 1), participants were presented with 267 words (100 critical words and 167 filler words). In each trial, one word and one circle (red, yellow, or green) appeared simultaneously at the center of the screen for 100 ms, with a vertical distance of 3 cm between them, after which the circle was removed and the word continuously shown for another 400 ms. There was a 500 ms inter-stimulus interval between successive trials. The study list was organized into 40 blocks of five words each, with no interruption between successive trials. Half of the blocks were assigned to the Cue Condition and the other half to the No-Cue Condition. Critical words encoded with a red circle (target-paired words) were always placed in the third position of each block. Critical words encoded with a green circle (distractor-paired words) were randomly located either in the first or fifth position of each block. Critical words encoded with a yellow circle (cue-paired words) were always placed in the position prior to the target words, the second position under the Cue Condition. All other words in the blocks (non-critical) were paired with a green circle. To reduce the regularity of target presentation, one to three filler words (67 words paired with a green circle) were randomly interspersed between two consecutive blocks, and other filler words (100 words paired with a green circle) were inserted in the remaining positions within each block. For example, if the first and third positions are placed with a distractor word and a target word under the No-Cue Condition, then the second, fourth, and fifth positions were placed with filler words. Thus, sixty of one hundred words were used as filler words in the No-Cue Condition (3*20) while forty filler words were in the Cue Condition (2*20). Participants were instructed to read the words and rapidly press the space bar key when they detected a target red circle below the word, but they weren’t required to memorize the words. They were also told that a cue represented the arrival of an incoming target.

The encoding phase was followed by a test phase, in which participants completed a word recognition test. A total of 120 words were presented at the center of the screen. This included 100 previously studied items (20 target-paired words under the Cue Condition, 20 distractor-paired words under the Cue Condition, 20 cue-paired words under the Cue Condition, 20 target-paired words under the No-Cue Condition, and 20 distractor-paired words under the No-Cue Condition) and 20 previously unstudied words. Participants were instructed to press two keys to indicate whether the word had been shown in the encoding phase: the *F* key for positive and *J* key for negative.

The experiment’s procedure was programmed in Presentation 1.0 and run on a DELL Dimension 8200 computer. All stimuli were presented centrally on a 7.8 cm × 9 cm white pane that was displayed on the computer monitor center against a black background. All stimuli were shown in white. The distance between the eyes and monitor was approximately 80 cm.

### Results

#### Detection task performance

The hit rate for target detection under the Cue Condition (88.7% ± 2.2) was significantly higher than for the No-Cue Condition (72.7% ± 2.9), *t* (25) = 5.23, *p* < 0.001, *d* = 1.03, 95% CI = (0.54, 1.50). The mean false alarm rates (i.e., mean% of incorrect space bar presses during encoding phase) for distractors and cues under the Cue Condition were 0.13% and 12.35%, respectively. The mean false alarm rate for distractors under the No-Cue Condition was 0.23%. The response time to the target under the Cue Condition (146 ± 14 ms) was significantly lower than under the No-Cue Condition (302 ± 5 ms), *t* (25) = 12.08, *p* < 0.001, *d* = 2.36, 95% CI = (1.60, 3.11).

#### Recognition task performance

**FIGURE 2:**
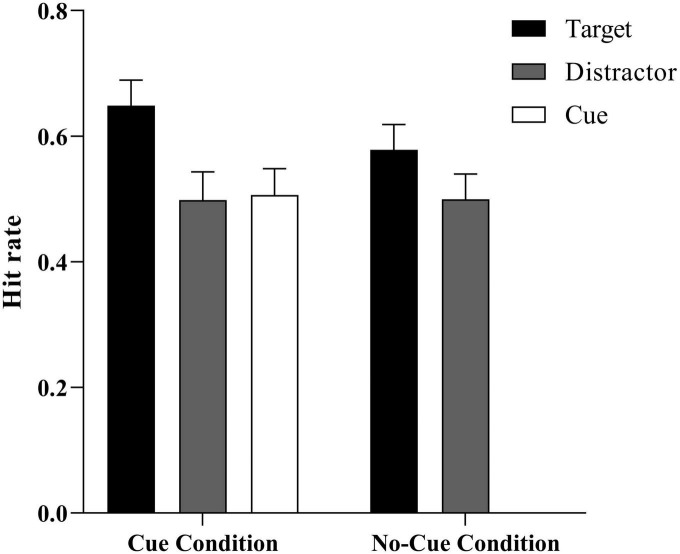
Mean hit rate as a function of stimulus type (target vs. distractor vs. cue) × condition (cue vs. no-cue). Error bars represent ± 1 *SE* of the mean.

We examined whether a participant correctly identified that a word had been presented (positive) or not (negative) in the previous encoding phase (see Table 1 and Figure 2). A separate *t*-test showed that participants performed better than the false alarm rate (0.25 ± 0.04) for words in each condition (all *t*s > 6.32, *p*s < 0.001), meaning that recognition judgments were not likely to be random guesses.

**TABLE 1:** Overall performance on word recognition test in Experiment 1.

Type of stimulus	Cue condition			No-Cue condition	
	**Target**	**Distractor**	**Cue**	**Target**	**Distractor**
Hit rate (%)	0.65 (0.04)	0.50 (0.05)	0.51 (0.04)	0.58 (0.04)	0.50 (0.04)
RT (ms)	918 (66)	895 (55)	887 (50)	995 (66)	955 (80)

*Standard errors are in parentheses.

To explore the influence of cues on ABE, an ANOVA 3 (trial type: distractor, cue, and target) × 2 (group: cue, no-cue) was conducted on the hit rate for Experiment 1. In the no-cue group, we took the mean value of the distractors that were shown in place of the cue. The results showed that the main effect of group was not significant, *F* (1, 50) = 3.66, *p* > 0.05. There was no significant difference between memory performances under the cue and no-cue conditions. The main effect of trial type was significant, *F* (2, 50) = 17.57, *p* < 0.001, η^2^_*p*_ = 0.41, 95% CI = (0.22, 0.53). The *post hoc* analysis found that the target-paired words were significantly better memorized than were the distractor- and cue-paired words, *F*_1_ (2, 24) = 16.15, *p*_1_ < 0.001, *F*_2_ (2, 24) = 13.07, *p*_2_ = 0.001. No difference was found between the distractor- and cue-paired words, *F* (2, 24) = 0.36, *p* > 0.05, BF_01_ = 6.13, indicating a moderate level of evidence supporting no statistically significant difference between the performances of distractor- and cue-paired words (Wagenmakers et al., 2017). The interaction effect of trial type and group was significant, *F* (2, 50) = 4.65, *p* = 0.04, η^2^_*p*_ = 0.16, 95% CI = (0.22, 0.53). A simple effect analysis showed that in the distractor-paired words, there was no significant difference between the cue and no-cue performances, *F* (1, 25) = 0.004, *p* > 0.05. However, in the target-paired words, the performance under the cue condition was significantly better than under the no-cue condition, *F* (1, 25) = 5.78, *p* = 0.02, BF_10_ = 2.26, indicating a moderate level of evidence supporting the conclusion that the performance of target-paired words in the cue condition was significantly better than in the no-cue condition.

We also conducted the same ANOVA on the RTs and did not find any significant differences among them (all *F*s < 2.59, *p*s > 0.05).

### Discussion

In Experiment 1, there was no significant difference in the memory performances of the cue- and distractor-paired words. This was contrary to the hypothesis that cues would lead to enhanced memorization of paired stimuli, and may indicate that if cues do not require corresponding responses, those events will not trigger ABE. That, in turn, may indicate the important role of responses in the production of ABE.

Memory performance of target-paired words under the cue condition was significantly better than under the non-cue condition (We excluded the data with false alarm rates to distractors and cues in the encoding phase greater than 0.1 and conducted similar data analyses for Experiment 1. The results were similar to that of the analysis in which all data were included, so the role of cues in ABE was not closely related to detection task performance in the encoding phase). That is, a larger ABE was found under the former rather than the latter, a result that agreed with our hypothesis. This means that the presence of a cue could provide a memory advantage in target-paired words, the first evidence that an alerting network may play an important role in ABE. Cues can trigger alertness networks that last 250 to 600 ms after the cue has disappeared (Weichselgartner and Sperling, 1987; Nakayama and Mackeben, 1989; Olivers and Meeter, 2008). We speculate that the enhancement effect of cues on ABE in Experiment 1 may have been due to the superimposed promotion effect of an alertness network on ABE. In other words, an alertness network may not be the key factor that triggers the production of ABE, but it could be a moderator of enhanced ABE.

There is another possibility regarding how cues enhanced ABE in Experiment 1. Specifically, although cues can trigger an alertness network, they can also enhance endogenous temporal orientation attention when the target appears. Different from the bottom-up exogenous temporal attention orientation directly triggered by external stimuli, endogenous temporal attention orientation is mainly triggered by predictive cues and is a top-down process (Coull and Nobre, 1998; Coull et al., 2000). Recent studies have found that ABE can be triggered by both exogenous and endogenous temporal attention orientation (Sisk and Jiang, 2020). Therefore, in Experiment 1, whether the enhancement effect of cues on ABE is due to the alerting effect triggered by those cues or an enhancement effect presented by the superposition of endogenous temporal attention orientation on the target stimulus which is triggered by the cues and exogenous temporal attention orientation triggered by target detection, the overall issue required further testing in Experiment 2.

## Experiment 2

### Experiment objective

Experiment 2 inserted a middle distractor between the cue and target, in order to extend the time interval between them. The middle distractor also allowed us to hypothesize that all processing of stimuli during the period of the alert state would be enhanced. If alerting could enhance all of the processing of stimuli during its effective time, then the correct recognition of middle distractor-paired words would be enhanced over other distractor-paired words.

To clarify the reason for the enhancement effect of cues on ABE that was found in Experiment 1, Experiment 2 extended the interval between the cue and target. This made the alerting effect of the cue disappear because that effect only lasts about 600 ms. This allowed for a determination of when the alertness effect of the cue disappeared, and if that would affect whether it enhanced the ABE. In Experiment 2, we hypothesized that if the cue does not enhance the ABE, the enhanced effect found in Experiment 1 was caused by its alertness effect. If the cue in Experiment 2 also enhanced the ABE, it would show that the enhancement effect seen in Experiment 1 was caused by the endogenous temporal attention orientation it induced and the superposition of the target.

### Materials and methods

#### Participants

Similar to Experiment 1, the average effect size used in previous studies (Mulligan et al., 2014; Mulligan and Spataro, 2015) of the ABE was *f* = 0.48; this served as the *a priori* effect size for the present research. G*Power 3.1 was employed to calculate that Experiment 2 required at least nine participants to achieve a statistical power of 0.95 in the repeated measures ANOVA (Faul et al., 2007). Twenty-five undergraduate students at Fujian Normal University, with a mean age of 21.8 years, participated in this experiment. All participants claimed to be in good health, with normal or corrected-to-normal vision, and no history of neurological illness. All gave their informed consent. As in the previous experiment, we included in Experiment 2 only those participants with less than 30% of responses to the cue. Consequently, three subjects were excluded. Thus, there were 22 participants in the experiment.

#### Design and materials

Attention conditions (Cue Condition vs. No-Cue Condition) were provided to each participant. A total of 287 critical words were selected from the Modern Chinese Frequency Dictionary Beijing Language College Language Instruction Institute (1986 edition): 140 critical words and 147 non-critical words. A total of 140 critical words were randomly and evenly divided into seven sets, with 20 words in each set. Six sets were assigned to two encoding conditions, with one set in each trial: target, distractor, middle distractor, and cue trials under the Cue Condition and target and distractor trials under the No-Cue Condition. The one remaining set was presented as new words for the recognition task. A total of 147 additional non-critical words were selected as fillers for the encoding phase.

#### Apparatus and procedure

**FIGURE 3:**
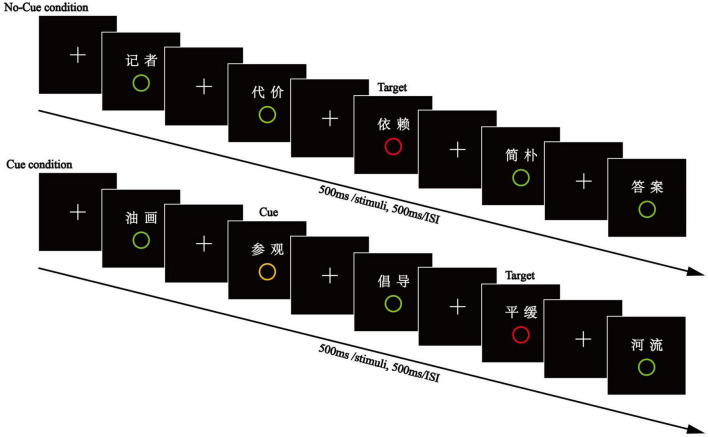
The encoding phase of Experiment 2. Between the cue- and target-paired words was inserted a word paired with a middle-distractor circle.

Experiment 2 was similar to Experiment 1, with the exception that a word paired with a middle-distractor circle was inserted between the cue- and target-paired words (see Figure 3). Participants were told that a (middle) distractor would appear after a cue, and then a target would follow; at that point, they should immediately press the space bar key. One hundred and forty words were presented in the test phase, including 120 studied items (20 target-paired words, 20 distractor-paired words, 20 middle distractor-paired, and 20 cue-paired words under the Cue Condition, 20 target-paired words and 20 distractor-paired words under the No-Cue Condition) and 20 unstudied words.

### Results

#### Detection task performance

Overall, the mean accuracy on the detection task was 79.7% ± 2.6% (between subjects standard error). Analyses of the accuracy (hit rate) for target detection indicated significantly better performance in the Cue Condition (84.1% ± 2.4%) than in the No-Cue Condition (75.2 ± 3.9%), *t* (21) = 2.19, *p* < 0.05, *d* = 0.48, 95% CI = (0.09, 0.90). The mean false alarm rates (i.e., mean% of incorrect space bar presses during encoding phase) for distractors, middle distractors, and cues under the Cue Condition were 0.12, 0.91, and 12.7%, respectively. The mean false alarm rate for distractors under the No-Cue Condition was 0.23%. Analysis of the response times showed significantly faster times for target detection in the Cue Condition (217 ± 20 ms) than in the No-Cue Condition (306 ± 6 ms), *t* (21) = 4.35, *p* < 0.001, *d* = 0.93, 95% CI = (0.42, 1.42).

#### Recognition task performance

**FIGURE 4:**
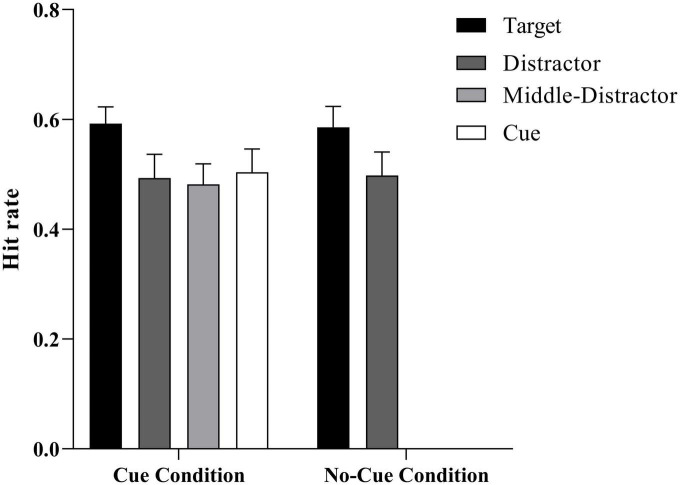
Mean hit rate as a function of the stimulus type (target vs. distractor vs. middle distractor vs. cue) × condition (cue vs. no-cue). Error bars represent ± 1 *SE* of the mean.

We examined whether a participant correctly identified if a word had previously been presented (positive) or not (negative) in the encoding phase (see Table 2 and Figure 4). Separate *t*-tests showed that participants performed better than the false alarm rate (0.28 ± 0.03) for words in each condition (all *t*s > 5.70, *p*s < 0.001), meaning that recognition judgments were not likely to be random guesses.

**TABLE 2:** Overall performance on word recognition test in Experiment 2.

Type of stimulus	Cue condition				No-Cue condition	
	**Target**	**Distractor**	**Middle distractor**	**Cue**	**Target**	**Distractor**
Hit rate (%)	0.59 (0.03)	0.49 (0.04)	0.48 (0.04)	0.50 (0.04)	0.59 (0.04)	0.50 (0.04)
RT (ms)	1,004 (48)	1,089 (92)	1,117 (74)	1,076 (71)	1,078 (74)	1,075 (56)

*Standard errors are in parentheses.

To explore the influence of cues on ABE, an ANOVA 4 (trial type: distractor, cue, middle, and target) × 2 (group: cue, no-cue) was conducted for the hit rate in Experiment 2. In the no-cue group, we took the mean values of the distractors that occurred at the cue position and the middle distractor for the no-cue group. The results showed that the main effect of group was not significant, *F* (1, 63) = 0.002, *p* > 0.05. There was no significant difference between the memory performances under the cue and no-cue conditions. The interaction effect of trial type and group was not significant, *F* (3, 63) = 0.05, *p* > 0.05. The main effect of trial type was significant, *F* (3, 63) = 7.72, *p* < 0.001, η^2^_p_ = 0.27, 95% CI = (0.22, 0.53). To investigate the ABE difference under various conditions in greater detail, a *post hoc* analysis was conducted of the cue and no-cue groups. In the no-cue group, the performance of target-paired words was significantly better than of distractor-paired words, *F* (3, 19) = 7.20, *p* = 0.002. In the cue group, the performance of target-paired words was significantly better than that of distractor-, middle distractor-, and cue-paired words, *F*_1_ (3, 19) = 27.85, *p*_1_ < 0.001, *F*_2_ (3, 19) = 11.19, *p*_2_ = 0.003, *F*_3_ (3, 19) = 27.89, *p*_3_ < 0.001. There was no significant difference in the performance of target-paired words between the cue and no-cue groups, *F* (1, 19) = 0.01, *p* = 0.83, BF_01_ = 4.39, indicating a moderate level of evidence supporting no statistically significant difference in the performance of cue- and no-cue target-paired words. ABE was evident in both the cue and no-cue conditions. However, despite the results indicating obvious ABE in both groups, no promoting effect of cues on ABE was found after the extended time interval, because the performance of cue-paired words was not significantly better than that of distractor-, middle distractor-paired words, *F*_1_ (3, 19) = 0.75, *p*_1_ > 0.05, *F*_2_ (3, 19) = 0.43, *p*_2_ > 0.05.

We also conducted the same ANOVA on the RTs and did not find any significant differences among them (all *F*s < 1.69, *p*s > 0.05).

### Discussion

In Experiment 2, there was no significant difference in memory performance for the cue- and distractor-paired words. This second experiment replicated the findings of Experiment 1 (We also excluded the data with false alarm rates to distractors and cues in the encoding phase greater than 0.1 and conducted similar data analyses for Experiment 2. The results were similar to that of the analysis in which all data were included, so the role of cues in ABE was not closely related to detection task performance in the encoding phase), showing that attentional cues do not trigger ABE if they do not require a response. In addition, Experiment 2 didn’t show any significant difference in memory performance between the cue- and distractor-paired words. These results may indicate the important role of responses in ABE production. Recent studies have also supported this view, claiming that responses are key to ABE production (Toh and Lee, 2022). Toh and Lee (2022) introduced the visual search paradigm, finding that when participants were asked to search for target-paired items without corresponding key responses, those items failed to show a memory advantage, suggesting that ABE was triggered by the response rather than target recognition. Therefore, the results of the present study not only show that not all attentional cues can produce ABE, they also serve as supplementary evidence of the key role of responses in the production of ABE.

More importantly, Experiment 2 did not find an enhancement effect of cues on ABE (as seen in Experiment 1), and there was no advantage for words paired with middle distractors relative to those paired with regular distractors. This indicates that when the interval between cue and target is extended, cues cannot enhance ABE. Therefore, after the alerting effect of the cue dissipated, there was no enhancement of ABE under the Cue Condition. Since the results of Experiment 2 (which included the endogenous orientation of the cue) ruled out the possibility that the enhancement of ABE in Experiment 1 was related to the cue’s endogenous orientation, a more reasonable explanation of the enhanced ABE in Experiment 1 was that an alerting process evoked by the cue was added to the ABE activated by the target. Both processes worked together to exaggerate ABE in the Cue Condition.

## General discussion

The current study used the ABE paradigm (Swallow and Jiang, 2012), but participants encoded words incidentally. We found that not all attentional cues lead to enhancement of the memory of concurrently paired words. By comparing the memory of target-paired words to that of distractor-paired words between the Cue and No-Cue Conditions, we found that the ABE existed in both, and using a cue promoted the ABE in the memory of target-paired words. However, such enhancement produced by cues only applied to target-paired words immediately presented after cue-paired words.

Neither of our experiments showed a memory advantage for cue-paired words (as compared to target-paired words), indicating that ABE requires a specific type of behaviorally relevant event stimulus (i.e., a target). One possible explanation is that ABE relies upon a behavioral response. According to the dual-task interaction model (DTI) described in Swallow and Jiang (2013), after items are detected, they are categorized into targets or distractors based on how the person’s behavior is guided. The central executive gives priority to processing target stimuli, leading to the production of an overt or covert response (e.g., a planned motor response or counting); this, in turn, triggers a temporally selective attention mechanism (Olivers and Meeter, 2008), potentially due to a transient increase in norepinephrine released from the locus coeruleus (Aston-Jones and Cohen, 2005). It therefore enhances the processing of all perceptual information present alongside the target. Toh and Lee (2022) used a visual search task to investigate the attentional boost effect, changing the simple target detection task in the classical ABE paradigm and allowing participants to search for a target among various distractors. The target might or might not exist. Under different conditions, participants were asked to respond (*via* a keystroke/pressing a key or counting) in trials in which the target was either present or absent. The results showed that the memory performance for items responding to needs was better, whether the goal was present or not. Once again, the important role of the reaction in the production of ABE was emphasized. Based on this, Swallow et al. (2022) updated the DTI model, arguing that ABE originates in the decision to respond to a stimulus. The “decision to respond” is a form of engagement with the behaviorally relevant event and does not have to be an overt physical response like pressing a button. This means that in the present study, targets enabled the participants to make overt responses, whereas cues did not. In other words, behaviorally relevant events without accompanying responses will not produce ABE, illustrating the important role of responses in the production of ABE and offering supplementary evidence for Swallow et al. Namely, the existence of a response may have a significant impact on ABE. One could argue that a cue that does not require a response could be treated as a distractor. However, our Experiment 1 showed that participants exhibited a higher likelihood of detecting the target (i.e., the red circle) during encoding under the cue condition rather than the no-cue condition. This shows that different from a distractor, a cue plays an alerting role, and hence improves the performance of detecting the target after the cue. Participants were more likely to press the button in response to a cue trial than to a distractor trial, also suggesting different types of processing between them (Maki and Mebane, 2006).

The important thing is that Experiment 1 found that compared to distractor-paired words, target-paired words were better memorized under the cue condition than the no-cue condition, confirming our hypothesis that a cue enhances ABE. This cue-related effect may have been caused by the activation of an attentional alerting system or endogenous attentional orientation of the cue. To understand how a cue’s enhancement of ABE works, Experiment 2 was conducted to investigate if a cue still had a beneficial effect on ABE when the time interval between the cue and target was extended, thus excluding the alertness effect of the cue. The cue in Experiment 2 still having an enhanced effect on ABE would indicate that the production of ABE was not due to the alerting effect of the cue. Conversely, if the cue in Experiment 2 did not enhance ABE, this would indicate that the enhanced effect on ABE was due to the cue’s alerting effect, rather than endogenous attentional orientation. Ultimately, when the interval between the cue and target was extended, no enhancement effect of the cue on ABE was found. This result suggest that the enhancement effect of the cue on ABE in Experiment 1 was not a result of the endogenous attentional orientation effect of the cue, and instead the product of the superposition of the cue’s alertness and target promotion effects. Thus, we speculate that although the alerting network triggered by the cue did not involve a behaviorally relevant response, the attentional alerting network could reach a superposition and promote ABE, and thus serve as a regulating factor in ABE enhancement.

Alerting is an important attentional function that keeps the brain awake to process the priority. A number of studies have examined the relationship between the alerting system and norepinephrine pathways produced in the locus coeruleus (Posner and Petersen, 1990; Fan et al., 2005; Gabay et al., 2011; Posner, 2012). For example, Fan et al. (2005) combined the attention network test and event-related functional magnetic resonance imaging (fMRI) to explore the brain activity of alerting in relation to attention. In their experiments, under the cue condition, a cue appeared for 200 ms prior to a target, while it took a variable time interval (300–11,800 ms) for the subjects to press a button. An event-related fMRI was then conducted to record the brain activities under the cue and no-cue conditions. The results showed a fronto-parietal activation, along with the thalamus. According to Morrison and Foote (1986), the posterior parietal lobe, pulvinar, and superior colliculus are most innervated by the NE pathway. Recently, some research has also provided direct evidence of LC involvement in ABE production. For example, Moyal et al. (2022) found that compared with no-distractor tones or no tones, auditory target tones increased BOLD activity in LC regions defined using neuromelanin imaging. Yebra et al. (2019) further demonstrated that activation of brainstem voxels consistent with LC was significantly associated with ABE. These findings support that the LC-NE system provides the basis for maintaining alertness, which may explain why in the present research, the cue condition resulted in a higher likelihood of detecting the target (red circle) during encoding than did the no-cue condition. The occurrence of a cue allows subjects to enter a more alert state prior to a target (Fan et al., 2005), thus promoting the secretion of norepinephrine from the locus coeruleus. As mentioned above, the increase of norepinephrine may lead to a broad perceptual enhancement, which may boost the encoding of target-paired words.

In addition, Experiment 2 showed that an insertion of a middle distractor after a cue to delay the occurrence of a target promoted no enhancement in the memory of target-paired words, as compared with the No-Cue Condition. This shows that the alerting effect caused by a cue is time-limited. Nakayama and Mackeben (1989) similarly found that a cue could promote the processing of perceptual information related to a target. However, such enhancement only lasted for 250 to 600 ms after the onset of the cue (Weichselgartner and Sperling, 1987; Nakayama and Mackeben, 1989; Olivers and Meeter, 2008). Hence, beyond this period, the cue resulted in no alerting benefit for the performance of target-identification. In Experiment 2, there was a period of 1,500 ms between the cue and target, which could have reduced the enhancement of the cue in processing the target-paired words. In theory, when a subject observes a middle distractor after a cue, they should still be under an alerting state, and thus should result in enhanced memory of middle distractor-paired words, due to the increased release of norepinephrine during this period. Yet, our study found that the recognition accuracy for words presented with middle distractors was similar to that of words paired with distractors. This result indicates that although an alerting cue enhanced the encoding process of target-paired words, as mentioned above, this was due to the superposition effect of the cue and ABE overlapping. However, this effect may also have been limited by the acting time of ABE (100 ms) and could not enhance the memory performance of the middle distractor words. This also provides indirect evidence that several other attentional mechanisms may be involved in the ABE besides the alerting network, a topic that requires further investigation.

## Conclusion

In conclusion, the results of the current study show that not all task-relevant events (at least in terms of alerting cues) promote the memory of concurrently paired stimuli, but a cue can enhance the memory of target-paired stimuli. This indicates that cues can enhance ABE, and the effect is related to the attention alerting system. This, in turn, is connected to the alertness network triggered. In other words, the attention alerting system plays a certain regulatory role in the production of ABE. However, the effect only lasts for a limited range of time.

## Data availability statement

The raw data supporting the conclusions of this article will be made available by the authors, without undue reservation.

## Ethics statement

The studies involving human participants were reviewed and approved by School of Psychology at Fujian Normal University. The patients/participants provided their written informed consent to participate in this study.

## Author contributions

FH, GL, YM, YL, and SZ designed the research and reviewed the manuscript. FH, GL, and YL performed the research and analyzed the data. FH and GL wrote the manuscript text. YM provided critical comments. SZ provided important intellectual assistance in manuscript revision process. All authors contributed to the article and approved the submitted version.

## References

[B1] Aston-Jones G. Cohen J. D. (2005). An integrative theory of locus coeruleus-norepinephrine function: adaptive gain and optimal performance. *Ann. Rev. Neurosci.* 28 403–450. 10.1146/annurev.neuro.28.061604.135709 16022602

[B2] Beijing Language College Language Instruction Institute (1986). *Modern Chinese Frequency Dictionary (in Chinese).* Beijing: Beijing Language College Press.

[B3] Bouret S. Sara S. J. (2005). Network reset: a simplified overarching theory of locus coeruleus noradrenaline function. *Trends Neurosci.* 28 574–582. 10.1016/j.tins.2005.09.002 16165227

[B4] Coull J. T. Frith C. D. Büchel C. Nobre A. C. (2000). Orienting attention in time: Behavioural and neuroanatomical distinction between exogenous and endogenous shifts. *Neuropsychologia* 38 808–819. 10.1016/S0028-3932(99)00132-3 10689056

[B5] Coull J. T. Nobre A. C. (1998). Where and when to pay attention: The neural systems for directing attention to spatial locations and to time intervals as revealed by both PET and fMRI. *J. Neurosci.* 18 7426–7435. 10.1523/JNEUROSCI.18-18-07426.1998 9736662 PMC6793260

[B6] Davidson M. C. Marrocco R. T. (2000). Local infusion of scopolamine into intraparietal cortex slows covert orienting in rhesus monkeys. *J. Neurophysiol.* 83 1536–1549. 10.1152/jn.2000.83.3.1536 10712478

[B7] Dux P. E. Marois R. (2009). How humans search for targets through time: a review of data and theory from the attentional blink. *Attent. Percept. Psychophys.* 71:1683. 10.3758/app.71.8.1683 19933555 PMC2915904

[B8] Egeth H. E. Yantis S. (1997). Visual attention: Control, representation, and time course. *Ann. Rev. Psychol.* 48 269–297. 10.1146/annurev.psych.48.1.269 9046562

[B9] Fan J. McCandliss B. D. Fossella J. Flombaum J. I. Posner M. I. (2005). The activation of attentional networks. *Neuroimage* 26, 471–479. 10.1016/j.neuroimage.2005.02.004 15907304

[B10] Fan J. McCandliss B. D. Sommer T. Raz A. Posner M. I. (2002). Testing the efficiency and independence of attentional networks. *J. Cogn. Neurosci.* 14 340–347. 10.1162/089892902317361886 11970796

[B11] Faul F. Erdfelder E. Lang A.-G. Buchner A. (2007). G*Power 3: A flexible statistical power analysis program for the social, behavioral, and biomedical sciences. *Behav. Res. Methods* 39 175–191. 10.3758/bf03193146 17695343

[B12] Fossella J. Sommer T. Fan J. Wu Y. Swanson J. M. Pfaff D. W. (2002). Assessing the molecular genetics of attention networks. *BMC Neurosci.* 3:14. 10.1186/1471-2202-3-14 12366871 PMC130047

[B13] Gabay S. Pertzov Y. Henik A. (2011). Orienting of attention, pupil size, and the norepinephrine system. *Attent. Percept. Psychophys.* 73 123–129. 10.3758/s13414-010-0015-4 21258914

[B14] Kinchla R. A. (1992). Attention. *Ann. Rev. Psychol.* 43 711–742. 10.1146/annurev.ps.43.020192.003431 1539951

[B15] Lassiter G. D. Stone J. I. Rogers S. L. (1988). Memorial consequences of variation in behavior perception. *J. Exp. Soc. Psychol.* 24 222–239. 10.1016/0022-1031(88)90037-6

[B16] Leclercq V. Seitz A. R. (2012). Enhancement from targets and suppression from cues in fast task-irrelevant perceptual learning. *Acta Psychol.* 141 31–38. 10.1016/j.actpsy.2012.05.005 22842471

[B17] Lin J. Y. Pype A. D. Murray S. O. Boynton G. M. (2010). Enhanced memory for scenes presented at behaviorally relevant points in time. *PLoS Biol.* 8:e1000337. 10.1371/journal.pbio.1000337 20305721 PMC2838752

[B18] Maki W. S. Mebane M. W. (2006). Attentional capture triggers an attentional blink. *Psychon. Bull. Rev.* 13 125–131. 10.3758/bf03193823 16724779

[B19] Marrocco R. T. Witte E. A. Davidson M. C. (1994). Arousal systems. *Curr. Opin. Neurobiol*. 4, 166–170. 10.1016/0959-4388(94)90067-1 7913640

[B20] Morrison J. H. Foote S. L. (1986). Noradrenergic and serotoninergic innervation of cortical, thalamie and tectal visual structures in old and new world monkeys. *J. Comp. Neurol.* 243 117–128. 10.1002/cne.902430110 3950077

[B21] Moyal R. Turker H. B. Luh W. M. Swallow K. M. (2022). Auditory target detection enhances visual processing and hippocampal functional connectivity. *Front. Psychol.* 13:891682. 10.3389/fpsyg.2022.891682 35769754 PMC9234495

[B22] Mulligan N. W. Spataro P. (2015). Divided attention can enhance early-phase memory encoding: The attentional boost effect and study trial duration. *J. Exp. Psychol.* 41 1223–1228. 10.1037/xlm0000055 25181494

[B23] Mulligan N. W. Spataro P. Picklesimer M. (2014). The attentional boost effect with verbal materials. *J. Exp. Psychol.* 40 1049–1063. 10.1037/a0036163 24611436

[B24] Nakayama K. Mackeben M. (1989). Sustained and transient components of focal visual attention. *Vis. Res.* 29 1631–1647. 10.1016/0042-6989(89)90144-2 2635486

[B25] Olivers C. N. L. Meeter M. (2008). A boost and bounce theory of temporal attention. *Psychol. Rev.* 115 836–863. 10.1037/a0013395 18954206

[B26] Pashler H. (1994). Dual-task interference in simple tasks: Data and theory. *Psychol. Bull.* 116 220–244. 10.1037/0033-2909.116.2.220 7972591

[B27] Posner M. I. (2012). Attentional networks and consciousness. *Front. Psychol.* 3:64. 10.3389/fpsyg.2012.00064 22416239 PMC3298960

[B28] Posner M. I. Petersen S. E. (1990). The attention system of the human brain. *Ann. Rev. Neurosci.* 13 25–42. 10.1146/annurev.ne.13.030190.000325 2183676

[B29] Schwan S. Garsoffky B. (2004). The cognitive representation of filmic event summaries. *Appl. Cogn. Psychol.* 18 37–55. 10.1002/acp.940

[B30] Sisk C. A. Jiang Y. V. (2020). The yellow light: Predictability enhances background processing during behaviorally relevant events. *J. Exp. Psychol.* 46 1645–1658. 10.1037/xlm0000838 32271063

[B31] Swallow K. M. Broitman A. W. Riley E. Turker H. B. (2022). Grounding the attentional boost effect in events and the efficient brain. *Front. Psychol.* 13:892416. 10.3389/fpsyg.2022.892416 35936250 PMC9355572

[B32] Swallow K. M. Jiang Y. V. (2010). The attentional boost effect: transient increases in attention to one task enhance performance in a second task. *Cognition* 115 118–132. 10.1016/j.cognition.2009.12.003 20080232 PMC2830300

[B33] Swallow K. M. Jiang Y. V. (2011). The role of timing in the attentional boost effect. *Atten. Percept. Psychophys* 73 389–404. 10.3758/s13414-010-0045-y 21264720

[B34] Swallow K. M. Jiang Y. V. (2012). Goal-relevant events need not be rare to boost memory for concurrent images. *Atten. Percept. Psychophys.* 74 70–82. 10.3758/s13414-011-0227-2 22012240

[B35] Swallow K. M. Jiang Y. V. (2013). Attentional load and attentional boost: a review of data and theory. *Front. Psychol.* 4:274. 10.3389/fpsyg.2013.00274 23730294 PMC3657623

[B36] Swallow K. M. Jiang Y. V. (2014). Perceptual load and attentional boost: a study of their interaction. *J. Exp. Psychol.* 40 1034–1045. 10.1037/a0035312 24364707

[B37] Swallow K. M. Zacks J. M. Abrams R. A. (2009). Event boundaries in perception affect memory encoding and updating. *J. Exp. Psy-chol. Gen.* 138 236–257. 10.1037/a0015631 19397382 PMC2819197

[B38] Toh Y. N. Lee V. G. (2022). Response, rather than target detection, triggers the attentional boost effect in visual search. *J. Exp. Psychol.* 48 77–93. 10.1037/xhp0000977 35073145

[B39] Wagenmakers E. J. Love J. Marsman M. Jamil T. Ly A. Verhagen J. (2017). Bayesian inference for psychology. Part II: Example applications with JASP. *Psychon. Bull. Rev.* 25 58–76. 10.3758/s13423-017-1323-7 28685272 PMC5862926

[B40] Weichselgartner E. Sperling G. (1987). Dynamics of automatic and controlled visual attention. *Science* 238 778–780. 10.1126/science.3672124 3672124

[B41] Yebra M. Galarza-Vallejo A. Soto-Leon V. Gonzalez-Rosa J. J. de Berker A. O. Bestmann S. (2019). Action boosts episodic memory encoding in humans via engagement of a noradrenergic system. *Nat. Commun.* 10 1–12. 10.1038/s41467-019-11358-8 31388000 PMC6684634

[B42] Zacks J. M. Speer N. K. Swallow K. M. Braver T. S. Reynolds J. R. (2007). Event perception: a mind - brain perspective. *Psychol. Bull.* 133 273–293. 10.1037/0033-2909.133.2.273 17338600 PMC2852534

